# Leydig‐like cells derived from reprogrammed human foreskin fibroblasts by CRISPR/dCas9 increase the level of serum testosterone in castrated male rats

**DOI:** 10.1111/jcmm.15018

**Published:** 2020-03-11

**Authors:** Hua Huang, Liang Zhong, Jin Zhou, Yanping Hou, Zhiyuan Zhang, Xiaoyu Xing, Jie Sun

**Affiliations:** ^1^ Department of Urology Shanghai Children’s Medical Center Shanghai Jiao Tong University School of Medicine Shanghai China

**Keywords:** CRISPR/dCas9, human fibroblasts, Leydig‐like cells, male hypogonadism, reprogramming, target gene activation

## Abstract

In the past few years, Leydig cell (LC) transplantation has been regarded as an effective strategy for providing physiological patterns of testosterone in vivo. Recently, we have successfully converted human foreskin fibroblasts (HFFs) into functional Leydig‐like cells (iLCs) in vitro by using the CRISPR/dCas9 system, which shows promising potential for seed cells. However, it is not known whether the reprogrammed iLCs can survive or restore serum testosterone levels in vivo. Therefore, in this study, we evaluate whether reprogrammed iLCs can restore the serum testosterone levels of castrated rats when they are transplanted into the fibrous capsule. We first developed the castrated Sprague Dawley rat model through bilateral orchiectomy and subsequently injected extracellular matrix gel containing transplanted cells into the fibrous capsule of castrated rats. Finally, we evaluated dynamic serum levels of testosterone and luteinizing hormone (LH) in castrated rats, the survival of implanted iLCs, and the expression levels of Leydig steroidogenic enzymes by immunofluorescence staining and Western blotting. Our results demonstrated that implanted iLCs could partially restore the serum testosterone level of castrated rats, weakly mimic the role of adult Leydig cells in the hypothalamic‐pituitary‐gonadal axis for a short period, and survive and secrete testosterone, through 6 weeks after transplantation. Therefore, this study may be valuable for treating male hypogonadism in the future.

## 1 INTRODUCTION

Male hypogonadism due to testosterone deficiency is common in men aged 40‐79 years, approximately 20% of whom are suffering from the disease, and incidence increases with age**.** 1, 2 Several studies show that hypogonadism can lead to arteriosclerosis, decreased bone mineral density and depressed mood as well as fatigue and sexual dysfunction.^3^, ^4^, ^5^ Testosterone replacement therapy (TRT) is capable of exerting some positive effects, such as improvement of muscle mass and sexual function^6^, ^7^; however, long‐term use of testosterone can also bring about a variety of side effects.^7^, ^8^, ^9^ Additionally, changes in normal testosterone levels in vivo show a physiological pattern,^10^ but it is difficult for TRT to achieve individualized requirements. Therefore, it is necessary to seek an alternative treatment with a physiological pattern for male hypogonadism.

In the last few years, Leydig cell (LC) transplantation has been regarded as an effective strategy for providing physiological patterns of testosterone in vivo.^11^ However, the key challenge is that an insufficient number of seed cells limits the application of cell transplantation because adult LCs lack the ability to proliferate, and the number of stem Leydig cells is extremely low.^12^, ^13^, ^14^

Several studies have demonstrated that functional Leydig‐like cells (iLCs) can be generated in a variety of ways, such as induced differentiation of stem cells in vitro or in vivo, adult cell reprogramming and so on.^15^, ^16^, ^17^ Our previous research revealed that stem cells have a low inducing efficiency in vivo or in vitro as well as ethical problems.^16^, ^18^ Recently, direct cell reprogramming seems to be a potential option for producing large numbers of seed cells via the direct and rapid conversion of cell fate.^17^, ^19^, ^20^, ^21^ Among reprogramming techniques, the CRISPR/dCas9 system, a new cell reprogramming tool, has emerged, acting through the activation of endogenous target genes, which is different from the forced expression of exogenous transcriptional factors.^22^, ^23^

In a previous study, we successfully reprogrammed human foreskin fibroblasts into functional iLCs by using the CRISPR/dCas9 SAM system for simultaneous activation of the endogenous target genes *Nr5a1*, *Gata4* and *Dmrt1,* and importantly, the iLCs possessed some characteristics of mature LCs, including the ability to secrete testosterone, respond to hCG in vitro and express Leydig steroidogenic enzymes.^12^ However, it is not known whether the reprogrammed iLCs can restore serum testosterone levels in vivo or mimic the role of adult Leydig cells in the hypothalamic‐pituitary‐gonadal axis. Moreover, there is a necessity to evaluate the survival and function of iLCs in vivo.

In the present study, we first converted the HFFs into functional iLCs and subsequently purified them. Next, we made a castrated animal model and injected extracellular matrix gel containing transplanted cells into the fibrous capsule of castrated rats. Finally, we demonstrated that the transplanted iLCs could partially restore the serum testosterone levels of castrated rats, mimic weakly the role of adult Leydig cells in the hypothalamic‐pituitary‐gonadal axis for a short period, and survive and secrete testosterone, though weakly, at 6 weeks after transplantation. Therefore, this study may be valuable for male hypogonadism in future.

## 2 MATERIALS AND METHODS

### 2.1 Cell culture

Primary human foreskin fibroblasts were isolated according to our previous study.^12^, ^15^ Briefly, after obtaining informed consent from the patient's guardian, foreskin tissues from a phimosis operation were collected according to the guidelines of the World Medical Association Declaration of Helsinki. The collected tissues were washed with phosphate buffer saline (PBS) containing 2% penicillin‐streptomycin (HyClone) three times. The subcutaneous tissue was removed, the remaining tissue subsequently was minced into 1‐2 mm pieces, and then the minced tissues were digested with collagenase type I solution (Slorbia) for 4 to 12 hours at 37°C.

The cells were filtered through a 70 μm cell strainer (Corning) and washed three times through centrifugation (800 rpm for 5 minutes), and the pellet was resuspended in αMEM (HyClone) containing 5% UltraGRO serum replacement (AventaCell BioMedical Co., Ltd.). The cells were plated in 10 cm dishes, and the culture medium was changed every 2 to 3 days.

### 2.2 Plasmid and lentivirus production

In the study, sgRNAs were designed and constructed according to our previous study.^12^ Three sgRNA sequences used for each target gene promoter in the study are listed in Table S1. The lenti‐sgRNA (MS2)‐Puro backbone was purchased from Genomeditech Co. Ltd., lenti‐MS2‐P65‐HSF_Hygro and lenti‐dCas9‐VP64_Blast were provided by Addgene (#61426 and #61425), and the Hsd3b promoter driving green fluorescent protein (EGFP) was cloned into the pCDH‐CMV‐MCS‐EF1‐Puro vector (TranSheep Bio); all of these constructs were confirmed by sequencing.

For lentiviral production, in brief, the lentiviral vectors and two homologous helper plasmids were cotransfected into 293T cells through the FuGENE® 6 transfection reagent (Promega). The supernatants containing virus were harvested 48 hours or 72 hours post‐transfection and then concentrated through a centrifugal ultrafiltration device (Amicon Ultra 15 mL 100 K, Millipore).

### 2.3 Establishment of stable cell lines and infection with sgRNAs

In the study, the stable Hsd3b‐dCas9‐MPH‐HFF cell line was established according to our previous study.^12^ Briefly, the cells were infected with the indicated concentrated lentiviral supernatants containing polybrene (10 μg/mL) (Santa Cruz) when primary HFFs grew to 80% confluence in 10‐cm dishes, and the culture supernatants were exchanged for fresh medium containing antibiotics 48 hours later. The antibiotic selection procedure was not less than 14 days, as shown in Figure S1B. The optimized concentrations for antibiotic selection were as follows: blasticidin S (30 μg/mL), puromycin (1.5 μg/mL) and hygromycin B (50 μg/mL) (Yeasen). After construction of the stable cell lines, the cells were infected with the corresponding lentiviruses encoding the sgRNA, and the lentivirus ratio was 1:1:1 at a multiplicity of infection of 20. Two days post‐infection, the culture supernatants were renewed.

### 2.4 Isolation of pre‐transplanted and post‐transplanted iLCs

The pre‐transplanted iLCs were sorted as described previously.^12^, ^17^ Briefly, 4 days post‐infection, cells were digested by 0.25% trypsin (Thermo Fisher Scientific), centrifuged (250× *g*, 5 minutes) and resuspended with 1× PBS. The iLCs were purified by a FACS instrument (MoFlo XDP, Beckman Coulter) based on green fluorescence.

To isolate post‐transplanted iLCs from Matrigel 6 weeks after transplantation, rats were killed humanely, and implanted Matrigel was extracted. The iLCs were isolated by Corning cell recovery solution (Discovery Labware, Inc) from extracted Matrigel according to the manufacturer's guidelines, and then these isolated cells were cultured in αMEM medium (HyClone) containing 5% UltraGRO serum replacement (AventaCell) for further morphologic analysis.

### 2.5 Castration and cell transplantation

In the study, eighty SPF‐grade male Sprague Dawley rats at 6 weeks of age were obtained from the Shanghai Model Organisms Center. All rats were kept under conditions with a controlled temperature of 25 ± 1°C, a light/dark cycle (12:12 hours) and relative humidity (45%‐55%). The standard rodent drinking water as well as diet was freely available. All surgical processes and postoperative care were approved by the Shanghai Children's Medical Center's Animal Care and Use Committee and conformed to the Guide for the Use and Care of Laboratory Animals. Eighty rats were randomly divided into the control group (20 rats), model group (20 rats), HFFs group (20 rats) and iLCs group (20 rats).

To evaluate whether iLCs could increase the serum testosterone level of castrated rats, castration models were made as described in previous studies.^24^, ^25^ In brief, first Sprague Dawley rats were anaesthetized using an intraperitoneal injection of sodium pentobarbital (40 mg/kg) and trachea intubation for artificial ventilation. The parenchymal tissue of bilateral testis was removed through the albuginea incision with rigorous hemostasis; furthermore, blood in the fibrous capsule was washed out by PBS, and the albuginea incision was sutured. Subsequently, a 1 mL volume of cold liquid Matrigel containing approximately 2 × 10^6^ transplanted cells was injected into the fibrous capsule of recipient testis, while, the model group was only injected with an equal amount of liquid Matrigel without cells. Testes or implanted Matrigel from all groups was examined at 1, 2, 3, 4, 5 and 6 weeks after cell transplantation.

### 2.6 Testosterone and luteinizing hormone measurement

Testosterone concentration was tested by chemiluminescence using the Access Testosterone Kit (REF 33 560, Beckman Coulter) as described in our previous study.^12^, ^18^ Briefly, serum or culture supernatants were collected at the indicated time‐points, and a 20‐μL aliquot of each sample was used for each determination. Measurements were automatically taken by the UniCel DxI 800 system (Beckman Coulter). The detection limits of this system are approximately 0.1‐16 ng/mL. The intra‐assay and inter‐assay variations were less than 5% and 10%, respectively.

To determine serum luteinizing hormone (LH) levels, a rat luteotropic hormone (LA) ELISA Kit (Mlbio) was used according to the manufacturer's instructions. Briefly, 50 μL of standard or sample was added in duplicate to the appropriate wells, and subsequently 100 μL of enzyme conjugate was added to standard wells and sample wells, except the blank well, and incubated for 1 hour at 37°C. After washing microtiter plates 4 times, 50 μL of substrate A and substrate B was added to each well, mixed gently and incubated for 15 minutes at 37°C. Furthermore, 50 μL of stop solution was added to each well, and the optical density was read at 450 nm by a microtiter plate reader within 15 minutes. This standard curve used to determine the amount in an unknown sample. Detection limits were 1.5 mIU/L‐48 mIU/L. The intra‐assay and inter‐assay variations were less than 10% and 15%, respectively. The test sensitivity is typically less than 0.1 mIU/mL.

### 2.7 Haematoxylin and eosin staining

To evaluate the implanted cells in grafts, histology staining was performed as previously described.^26^ Briefly, testes or implanted Matrigel from four groups of rats was collected and fixed in 4% paraformaldehyde (PFA) overnight at 4°C. Then, these fixed tissues were treated with a series of ethanol and xylene and then embedded in paraffin. Six micrometre (6 μm)‐thick cross sections were cut and dewaxed in distilled water for haematoxylin & eosin (H.E) staining. To enumerate the surviving cells, 10 random images per slice and six slices per rat were analysed by counting the number of cells per field of vision under ×200 magnification using ImageJ software (https://imagej.nih.gov/ij/).

### 2.8 Western blotting

At 1, 3 and 5 weeks after transplantation, the grafts (implanted Matrigel) or testicular tissues were subjected to protein extraction using RIPA lysis buffer (Solarbio) containing 1% protease inhibitors (Sangon Biotech) on ice for Western blotting analysis as previously described.^27^, ^28^ Protein concentrations were normalized by the Bradford method, and 40 μg of protein per channel was subjected to 10% SDS‐PAGE and subsequently transferred to polyvinylidene difluoride membranes (Millipore). The membranes were blocked with 5% skim milk for 1 hour at room temperature (RT) and incubated overnight at 4°C with primary antibodies: rabbit anti‐human CYP17A1 (1:500), HSD3B1 (1:1000), STAR (1:500), CYP11A1 (1:500) (Biorbyt) and GAPDH (1:2000) (Abcam). Then, the membranes were incubated with a goat anti‐rabbit antibody (1:5000) (Abcam) in the dark for 1 hour at RT, and corresponding bands were detected by an infrared laser imaging system (LI‐COR Odyssey). Protein expression levels were normalized to GAPDH by grayscale values using Image‐ProPlus 6.0 software (Media Cybernetics).

### 2.9 Immunofluorescence

Immunofluorescence staining was used to detect the expression of Leydig steroidogenic markers in grafts as previously described [dgRNA‐AAV]. Briefly, at the indicated time‐point after transplantation, fresh testes or grafts were harvested and hydrated in 30% sucrose overnight. Tissues were embedded in OCT (Sakura Tissue‐Tek) and quickly frozen in liquid nitrogen. Serial 12 μm sections were made by a Leica CM1520 system (Leica Biosystems Inc). Sections were rinsed 3 times with PBS for 5 minutes, incubated with blocking solution containing 3% BSA and 0.25% Triton X‐100 for 45 minutes, incubated with rabbit anti‐human CYP11A1 (1:100) (Abcam) and anti‐human CYP17A1 (1:50) (Abcam) overnight at 4°C, washed with 0.2% PBST, and subsequently incubated with a goat anti‐rabbit antibody conjugated to Alexa Fluor 647 (1:300) (Abcam) for 60 minutes at RT. After rinsing, the sections were stained with Hoechst 33 342 (Yeasen) and then examined using a fluorescence microscope (Leica DMi8).

### 2.10 Statistical analysis

Each experiment was performed independently at least three times, and the representative data are shown as the mean ± standard error of the mean (SEM). Statistical differences were evaluated by two‐tailed Student's *t* test or one‐way analysis of variance for more than two groups using Prism 6 software (GraphPad Software, Inc). A value of *P* < .05 was considered statistically significant.

## 3 RESULTS

### 3.1 Generation and purification of functional iLCs capable of secreting testosterone

**Figure 1:**
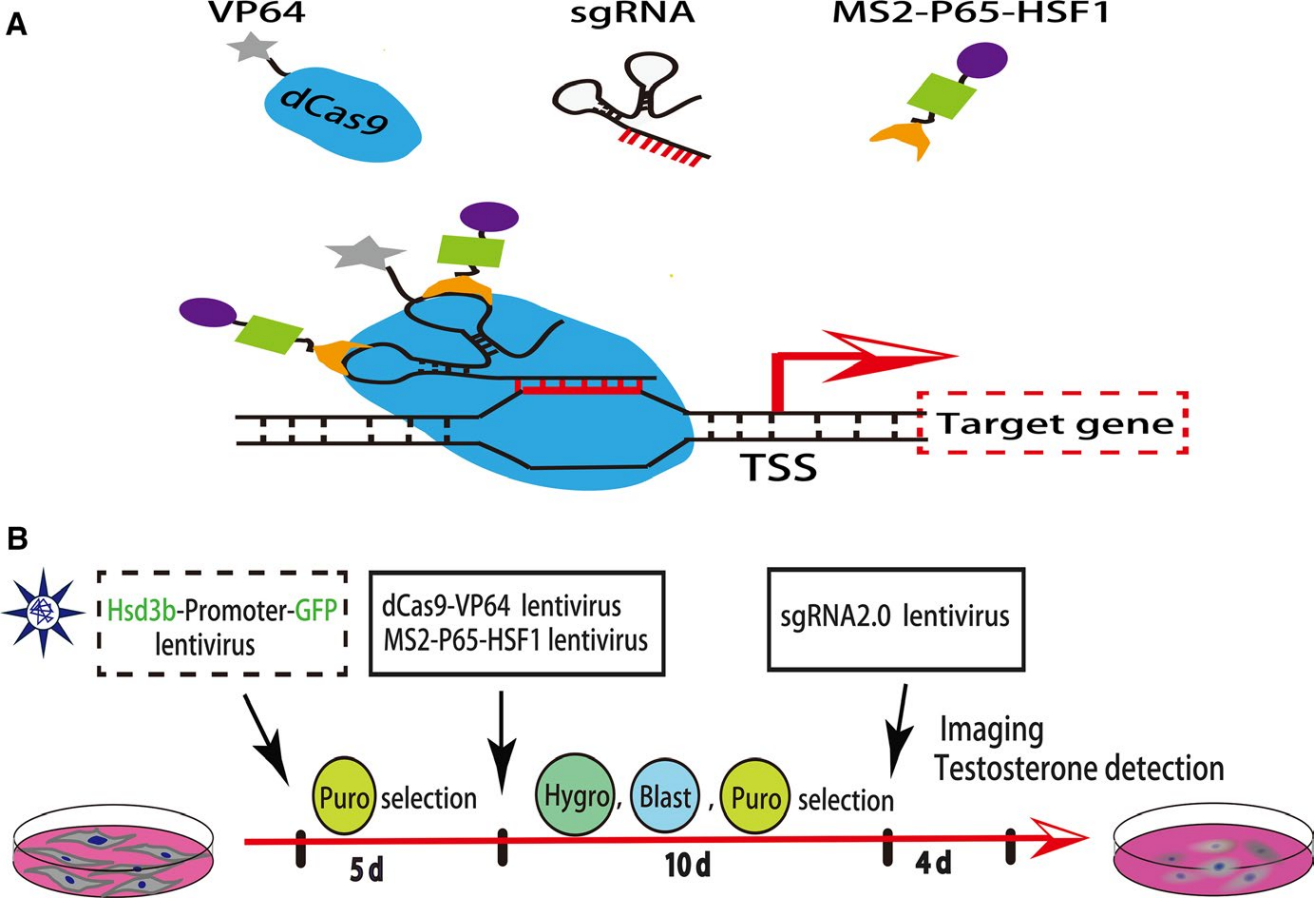
CRISPR/dCas9 SAM system and experimental procedures. A, Schematic of target gene activation through the CRISPR/dCas9 synergistic activation mediator (SAM) system. B, Summary schematic of the experimental procedures including infection of primary HFFs by lentiviruses and corresponding antibiotic selection in vitro

To achieve simultaneous activation of multiple endogenous genes including *Nr5a1*, *Gata4* and *Dmrt1*, in this study, we made use of the powerful second‐generation CRISPR/dCas9 SAM system (Figure 1A). First, primary human foreskin fibroblasts were infected with Hsd3b‐EGFP, dCas9‐VP64 and MS2‐P65‐HSF1 (SAM) lentiviruses and underwent multiple resistance screening (Figure 1B). After 15 days of resistance screening with puromycin, blasticidin S and hygromycin B, no green fluorescent protein (GFP) was detected, and stable Hsd3b‐dCas9‐MPH‐HFF cell lines were successfully constructed (Figure S1A). Subsequently, sgRNA lentiviruses targeting the Nr5a1, Gata4 and Dmrt1 genes were used to co‐infect the constructed cell lines, and Hsd3b:GFP‐positive cells were detected by fluorescence microscopy 4 days later (Figure S1B).

To obtain purified iLCs for in vivo transplantation, the Hsd3b:GFP‐positive cells were sorted by FACS based on green fluorescence (Figure S1C). The purified iLCs were further cultured for 4 days with serum‐free medium containing 5% serum replacement and hCG hormone (10 ng/mL) or not. The mean testosterone level of iLC supernatants was 1.48 ng/mL 4 days later, but adding hCG caused the iLCs to have a higher testosterone level with a mean value of 2.43 ng/mL (Figure S1D). Thus, this result suggested that these purified iLCs may be suitable for transplantation in vivo.

### 3.2 Transplantation of iLCs into the fibrous capsule of castrated rats and partial restoration of serum testosterone levels

**Figure 2:**
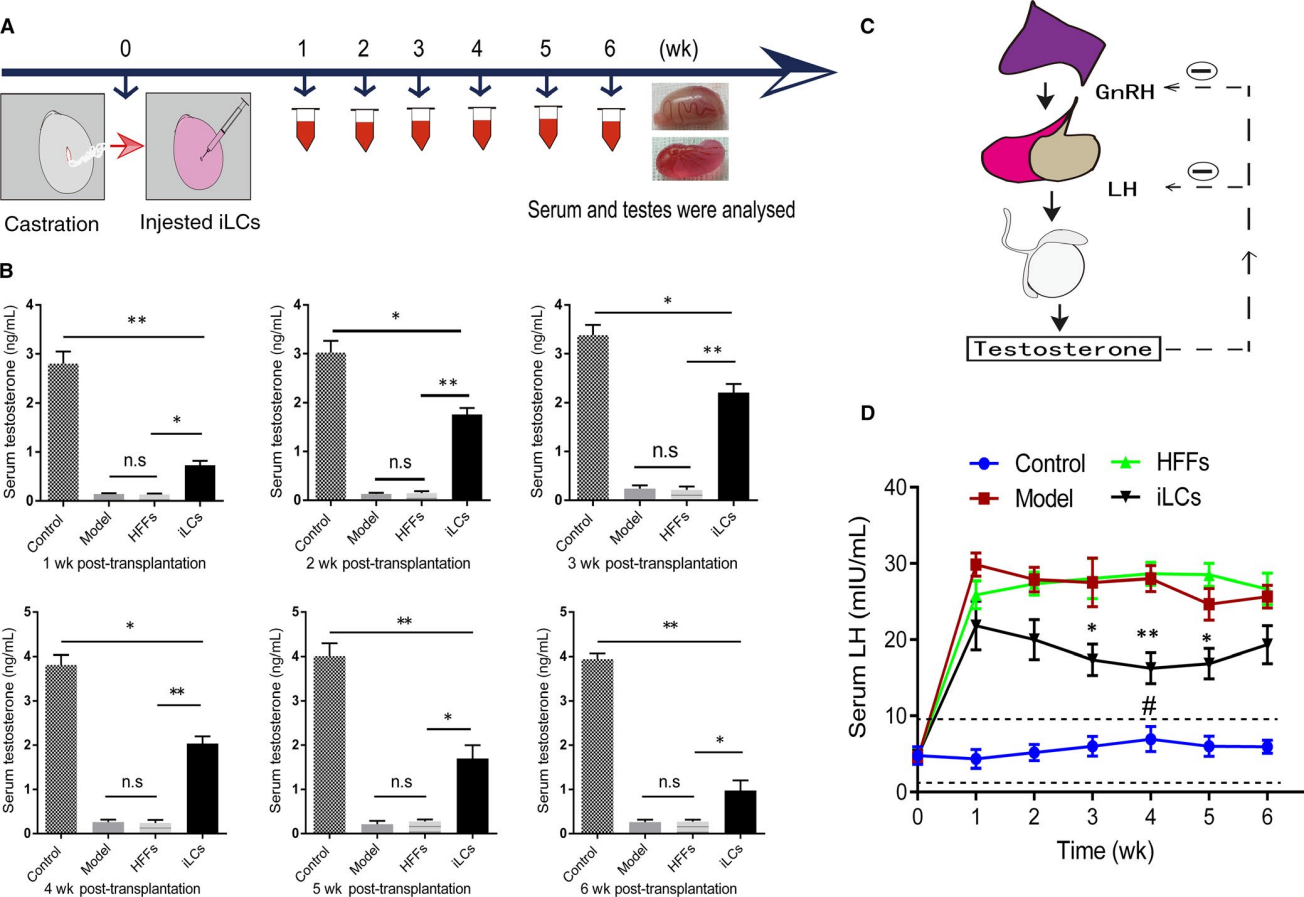
Transplanted iLCs accelerated the recovery of testosterone in castrated rats. A, Summary diagram for the experimental procedure in vivo. B, Testosterone levels were determined in serum at the indicated times after transplantation. C, Classic diagram showing the role of the hypothalamic‐pituitary‐gonadal axis in the production of testosterone in vivo. D, Luteinizing hormone (LH) levels were determined in serum at the corresponding times post‐transplantation. *P < .05, **P < .01 (iLCs vs HFFs or Model) and **^#^** *P* < .05 (iLCs vs Control), n = 3

To study whether the transplanted iLCs can produce testosterone in vivo and to observe restored degree of serum testosterone in castrated rats after cell transplantation, we injected 1 mL of extracellular Matrigel containing transplanted cells (2 × 10^6^/mL) into the fibrous capsule of castrated rats at the time of castrated animal model generation (Figure 2A). At 1, 2, 3, 4, 5 and 6 weeks after castration, the serum and testes were collected for analysis (Figure 2A). Consistent with our previous study, within a few days, the castrated animal model caused the serum testosterone level to decline significantly.^25^

The serum testosterone level of castrated rats with iLC implantation approached 1 ng/mL one week after implantation, while the serum testosterone level of the HFFs group was approximately 0.2 ng/mL, which was close to the level of the model group but significantly lower than that of the iLCs group. As the transplant time increased, the serum testosterone level of the iLCs group increased gradually and reached its peak (2.2 ng/mL), which recovered to approximately 56% of the level of control rats at 3 weeks after implantation and gradually declined 3 weeks later (Figure 2B).

The testosterone secretion function of Leydig cells is regulated by the hypothalamus‐pituitary‐gonad axis in vivo (Figure 2C). To further assess whether the transplanted iLCs could mimic the role of adult Leydig cells in the hypothalamic‐pituitary‐gonadal axis, we tested the levels of serum luteinizing hormone (LH) from rats of different groups. In the control group, serum LH levels were smoothly maintained between 2 and 9 mIU/mL from 1 week to 6 weeks after transplantation, which was lower than the other groups. For the iLCs group, however, the valley value of the serum LH level was approximately 15 mIU/mL, which was higher than that of the normal control but lower than those of both the HFFs and model groups, and moreover, the time‐point of the valley value occurred in the fourth week after transplantation, which was also the time‐point of the serum testosterone peak (Figure 2D). These results showed that transplanted iLCs could partially restore the serum testosterone level of castrated rats and weakly mimic the role of Leydig cells in the hypothalamic‐pituitary‐gonadal axis.

### 3.3 Most implanted iLCs are still alive and express steroidogenic enzymes of adult Leydig cells 6 weeks later

**Figure 3:**
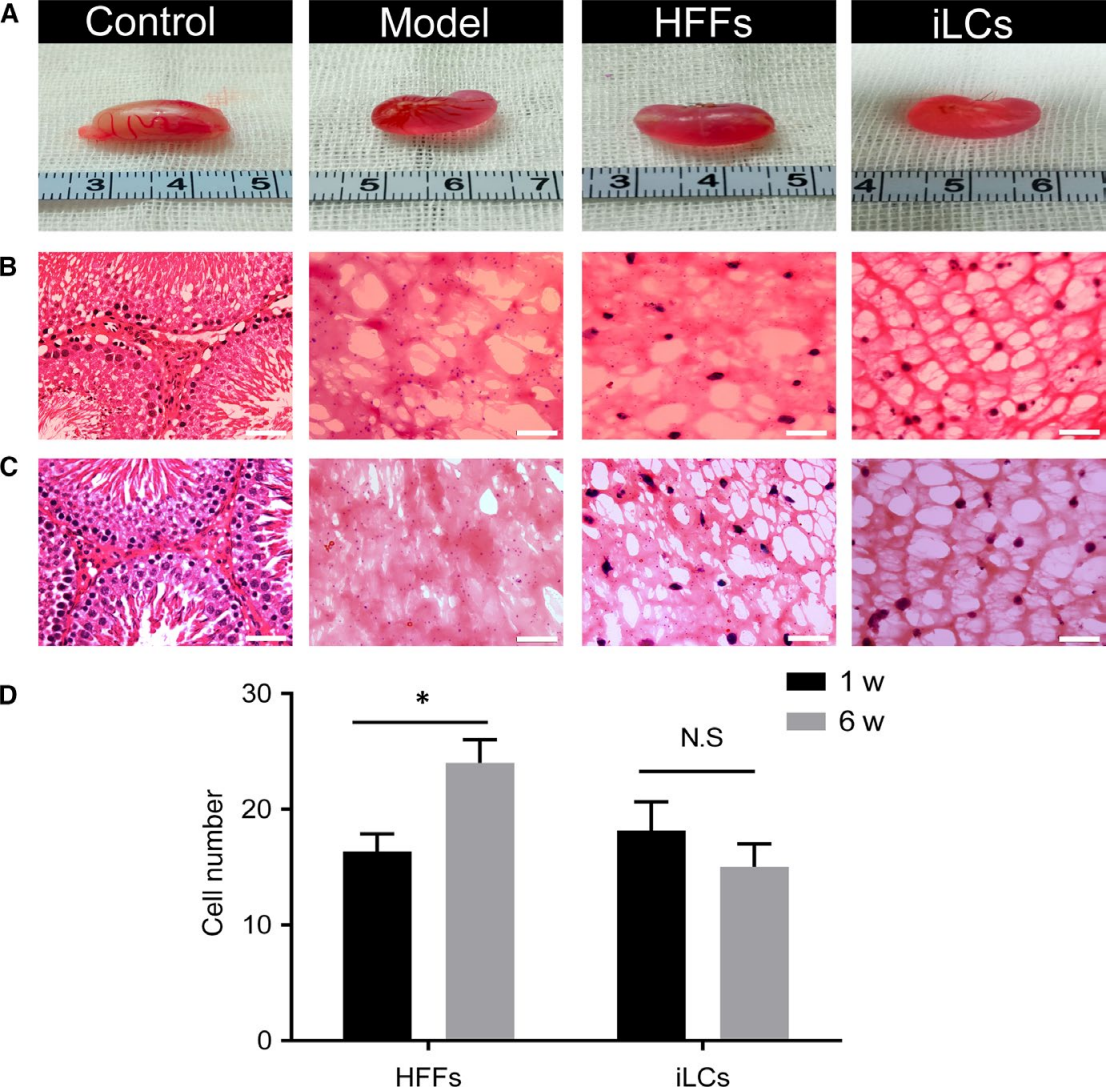
Survival of the transplanted cells in vivo 1 wk or 6 wk after transplantation. A, Representative photographs of macroscopic testis or Matrigel from four groups 6 weeks after transplantation. (B, C), Representative sections of testis or Matrigel from four groups 1 wk or 6 wk after transplantation stained with haematoxylin & eosin. Scale bar = 50 μm. D, Quantitative analysis of the transplanted cells in the Matrigel sections at the indicated time‐points. Five fields of vision per slide under ×200 magnification and five slides per Matrigel were counted (*P < .05, N.S: no statistical significance). Data are expressed as the mean ± SEM

To further observe the changes in the number and survival status of transplanted cells in vivo, testes or transplanted Matrigel from four groups of rats was collected (Figure 3A). Haematoxylin & Eosin (H.E) staining was performed on testes or transplanted Matrigel sections to observe the histomorphology at 1 week and 6 weeks after grafting. H.E staining showed that transplanted HFFs and iLCs were capable of surviving and distributed in the Matrigel just as Leydig cells localize in the interstitium of the testis, and the nuclei of HFFs were arranged in a spindle shape, but the nuclei of iLCs were arranged in a round‐like shape (Figure 3B,C).

**Figure 4:**
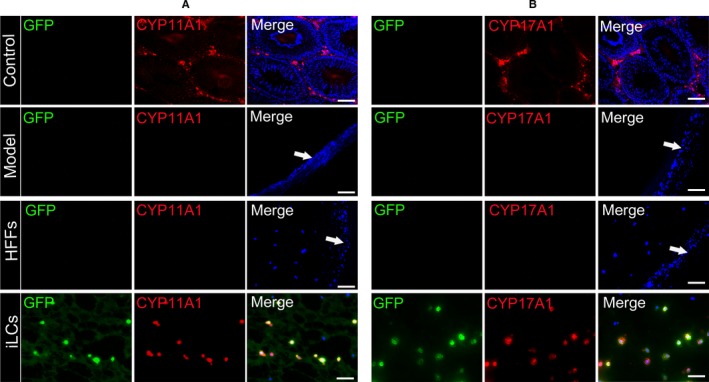
Transplanted iLCs expressed markers of adult Leydig cells in castrated rats 6 wk after transplantation. (A, B) Immunofluorescence staining showed the cells positive for Hsd3b:GFP (green) and CYP11A1 (red) or CYP17A1 (red) in the interstitial area of the testis or Matrigel 6 wk after implantation. The white arrows in this diagram point to albuginea of the testis. Nuclei were counterstained with Hoechst 33 342 (blue). Scale bar = 50 μm

Moreover, quantitative analysis showed that the number of living transplanted HFFs was greater at 6 weeks after grafting than at 1 week after grafting; however, the number of living transplanted iLCs did not change significantly (Figure 3D). It may be that to some extent, transplanted HFFs could still proliferate in vivo, while iLCs cannot. Meanwhile, immunofluorescence staining was performed on the testes or transplanted matrix gel sections at 6 weeks after transplantation to demonstrate whether the Hsd3b:GFP‐positive iLCs expressed steroidogenic enzymes of Leydig cells. Immunofluorescence staining showed that these transplanted iLCs expressed CYP11A1 and CYP17A1 6 weeks after grafting, but the HFFs and model groups did not (Figure 4A,B). The above results suggested that most transplanted iLCs could successfully adapt to the new niche, survive for at least 6 weeks and moreover express steroidogenic markers of Leydig cells.

### 3.4 Implanted iLCs in castrated rats show decreased protein expression of steroidogenic biomarkers 3 weeks after grafting

**Figure 5:**
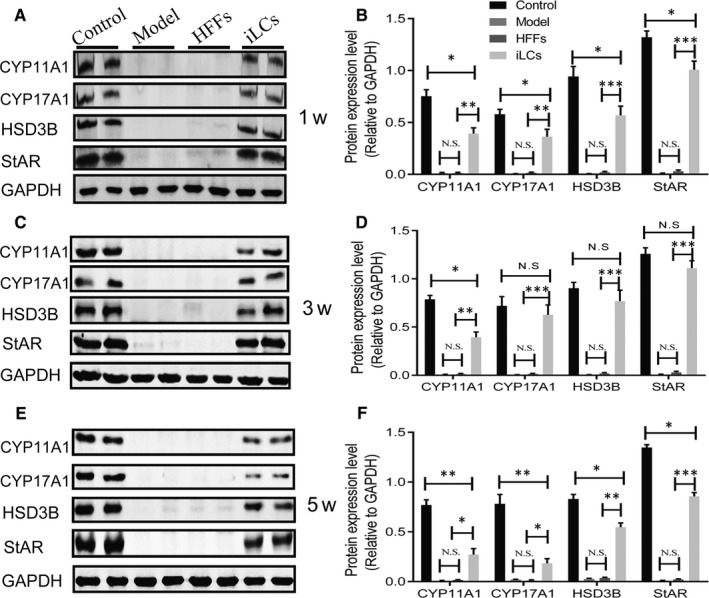
Protein expression levels of steroidogenic biomarkers at different time‐points after transplantation. (A, B) One week after transplantation, representative Western blotting for protein expression of CYP11A1, CYP17A1, StAR and HSD3B; relative protein expression levels were calibrated to GAPDH by grayscale values. (C, D), Three weeks after transplantation, Western blotting for protein expression of CYP11A1, CYP17A1, StAR and HSD3B; relative protein expression levels were calibrated to GAPDH by grayscale values. (E, F), Five weeks after transplantation, Western blotting for protein expression of CYP11A1, CYP17A1, StAR and HSD3B; relative protein expression levels were calibrated to GAPDH by grayscale values. All quantitative data are shown as the mean ± standard error of the mean, each group representing at least nine rats. *P < .05, **P < .01 and ***P < .001, N.S: no statistical significance

To further determine why testosterone levels begin to drop over 3 weeks after transplantation, expression levels of steroidogenic enzymes were determined at different time‐points after cell transplantation. In the study, Western blotting was performed on testicular tissue or implanted matrix gel containing HFFs or iLCs. The Western blotting assay showed that 1 week after grafting, the iLCs group in vivo expressed steroidogenic enzymes such as CYP11A1, CYP17A1, HSD3B and StAR, which are key for testosterone synthesis. In contrast to the control group, the iLCs group maintained a low expression level (Figure 5A,B). Inspiringly, at 3 weeks after grafting, the iLCs group strongly expressed these steroidogenic biomarkers, and in particular, the expression levels of HSD3B and StAR were close to those of the controls, while the model and HFFs groups demonstrated close to negative expression of these biomarkers (Figure 5C,D). Frustratingly, expression levels of these biomarkers in the iLCs group decreased significantly at 6 weeks after transplantation, and in particular, expression levels of CYP11A1, CYP17A1 and StAR were lower (Figure 5E,F). The results indicated that the expression levels of steroidogenic enzymes of the iLCs group in vivo showed a decreasing trend corresponding to the trend of serum testosterone levels of castrated rats receiving iLCs transplantation. Regarding this phenomenon, the new niche for the implanted iLCs may play a vital role.

### 3.5 iLCs isolated from grafts are still capable of producing testosterone

**Figure 6:**
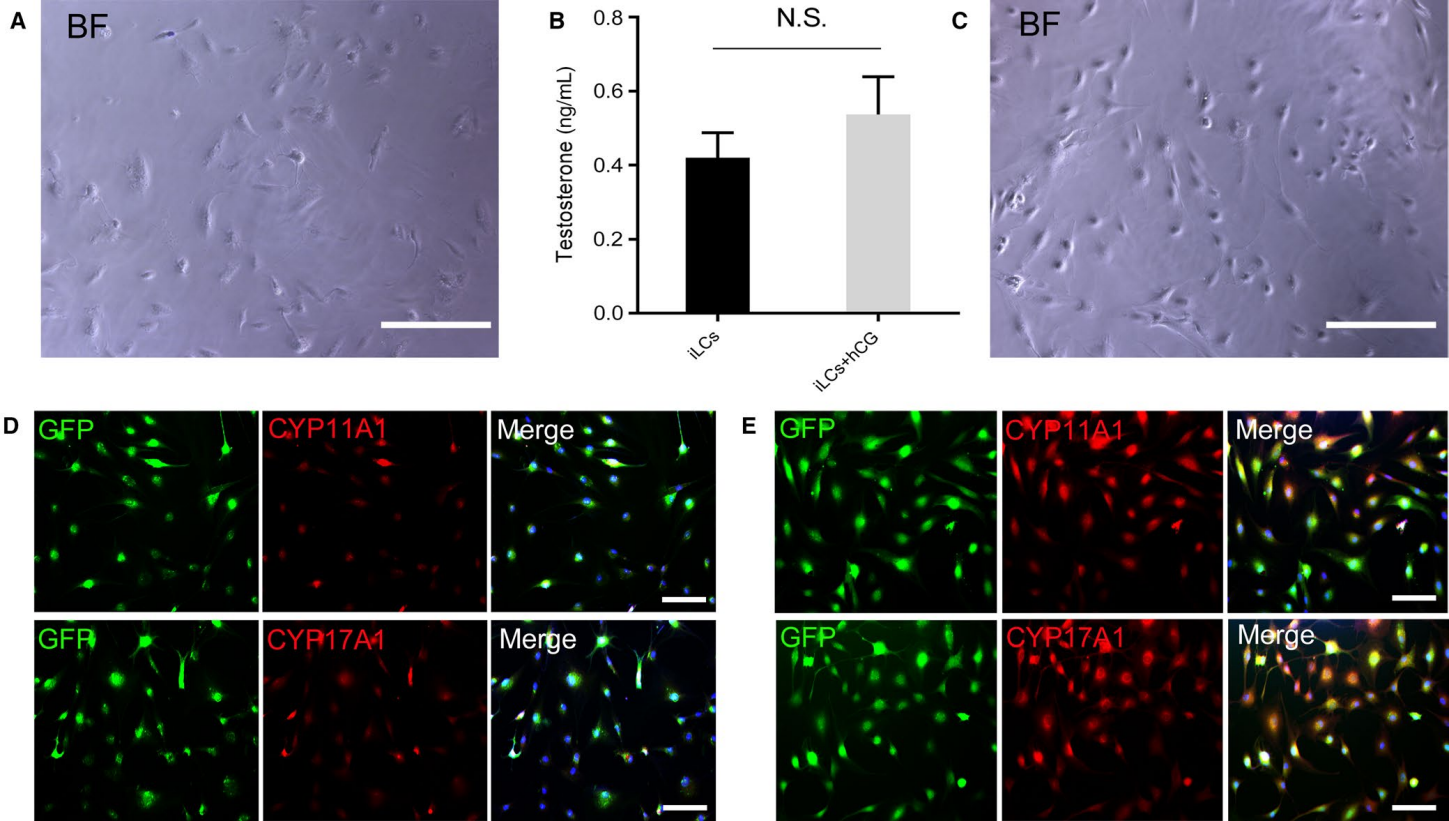
Post‐transplanted iLCs isolated from implanted Matrigel still possessed a few characteristics of Leydig cells. A, Representative photographs of isolated iLCs from the post‐transplantation Matrigel showed that cell morphology obviously changed compared with that of pre‐transplanted iLCs (Figure 6C). B, The isolated iLCs from Matrigel were still capable of producing testosterone. N.S: no statistical significance. C, Representative photographs of pre‐transplanted iLCs showing normal cell morphology. D, Representative immunofluorescence staining showed that the isolated iLCs from post‐transplantation Matrigel expressed low levels of Leydig steroidogenic markers. Scale bar = 100 μm. E, Representative immunofluorescence staining indicated pre‐transplantation iLCs highly expressed Leydig steroidogenic markers. Scale bar = 100 μm

To further compare the characteristics of implanted iLCs in vivo at 6 weeks after grafting with pre‐transplanted iLCs, the implanted iLCs were isolated by Corning cell recovery solution and seeded in 6‐well culture plates. The isolated cells were cultured for 3 days and showed abnormal cell size and shape compared with pre‐transplanted iLCs (Figure 6A,C). In addition, the iLCs isolated from the implanted Matrigel secreted testosterone weakly, and adding hCG failed to promote the secretion of testosterone compared with the pre‐transplanted iLCs (Figure 6B and Figure S1D).

Subsequently, to evaluate the expression levels of key steroidogenic enzymes in the iLCs, immunofluorescence staining was performed on the cells. Interestingly, the iLCs isolated from the transplanted Matrigel still expressed CYP11A1 and CYP17A1, though the expression levels of the markers were significantly decreased compared with those in pre‐transplanted iLCs (Figure 6D,E). The data indicate that with prolonged cell transplantation, the function of these cells gradually weakened, and cell ageing caused by adverse factors may be the main reason for this effect.

## 4 DISCUSSION

In our recent study, we successfully converted human foreskin fibroblasts into functional iLCs by simultaneous activation of the endogenous target genes *Nr5a1*, *Gata4* and *Dmrt1*, and to our knowledge, this is the first study of reprogrammed iLCs based on the CRISPR/dCas9 SAM system^12^; importantly, the iLCs showed some characteristics of adult LCs, including the ability to secrete testosterone, respond to hCG in vitro and express Leydig steroidogenic enzymes**.** In this study, we demonstrated that reprogrammed iLCs based on the CRISPR/dCas9 SAM system could survive for over 6 weeks and partially recover the serum testosterone level of castrated rats when they were transplanted into the fibrous capsule.

To obtain pre‐transplanted iLCs, we first isolated primary HFFs, further reprogrammed them into functional iLCs based on our previous method and purified the Hsd3b:GFP + cells by FACS.^17^, ^29^ In the study, cells were cultured with serum‐free medium containing 5% serum replacement, and we found that in terms of the reprogramming effect, the use of 5% serum replacement was superior to the use of 10% foetal bovine serum in our previous experiments.^12^ The pre‐transplanted iLCs could secrete testosterone and produce greater levels in vitro when hCG was added to the medium. To study whether the reprogrammed iLCs could restore serum testosterone levels in vivo or mimic the role of adult Leydig cells in the hypothalamic‐pituitary‐gonadal axis after transplantation, we generated a castrated animal model and injected Matrigel containing transplanted cells into the fibrous capsule of castrated rats. Consistent with our previous study, the serum testosterone level of the castrated rats decreased significantly within a few days.^25^ Interestingly, the testosterone concentration increased significantly a week after iLCs transplantation relative to the HFFs group and model group; however, in contrast to the control group, the concentration was significantly low. This result indicated that transplanted iLCs survived and began to work at the initial stage after transplantation. As the transplant time lengthened, the serum testosterone level of the iLCs group increased gradually and reached its peak (2.2 ng/mL) 3 weeks after implantation but gradually declined over 3 weeks later. Regarding this phenomenon, there may be many factors involved, such as off‐target effects despite low probability, the senescence of implanted cells caused by adverse factors in vivo, inflammatory reactions and so on, that eventually led to the observed result.^30^, ^31^, ^32^, ^33^ The trend of serum testosterone levels after iLCs transplantation was similar to our previous study in vitro.^12^, ^15^ Meanwhile, to evaluate whether transplanted iLCs could mimic the role of adult Leydig cells in the hypothalamic‐pituitary‐gonadal axis, we tested dynamic levels of serum luteinizing hormone (LH) from rats of different groups. In contrast to other time periods, the level of serum LH declined most significantly at 4 weeks after iLCs transplantation. Interestingly, it was also at this time that the testosterone concentration reached its highest level. However, the serum LH concentration persisted at a high level in the model and HFFs groups. The results suggested that implanted iLCs could mimic the partial role of adult Leydig cells in the hypothalamic‐pituitary‐gonadal axis, though weakly, in vivo.

To observe the survival status of transplanted cells in vivo, testes or transplanted Matrigel from four groups of rats was collected and analysed. It is well known that Matrigel containing a number of growth factors extracted from the mouse sarcoma acts as scaffold and nutrition for transplanted cells.^34^, ^35^, ^36^ In the present study, histological staining showed that implanted HFFs or iLCs were capable of surviving and distributed in the Matrigel just as Leydig cells localize in the interstitium of the testis. In addition, the nuclei of HFFs were arranged in a spindle shape, while the nuclei of iLCs were arranged in a round‐like shape. Interestingly, compared with one week after grafting, the number of living HFFs increased at 6 weeks after grafting, while the number of living transplanted iLCs did not change significantly. This may be because transplanted HFFs still have a certain degree of proliferative capacity in vivo.^37^ Meanwhile, Hsd3b:GFP‐positive iLCs still expressed steroidogenic enzymes of Leydig cells such as CYP11A1 or CYP17A1 at 6 weeks after transplantation, while the HFFs and model groups did not. The data indicated that most implanted iLCs could successfully adapt to the new niche, survive for at least 6 weeks and express steroidogenic markers of Leydig cells.

To further determine why testosterone levels began to drop over 3 weeks after transplantation, expression levels of steroidogenic enzymes were determined at different time periods after cell transplantation. One week after grafting, a Western blotting assay showed that the iLCs‐transplanted group moderately expressed steroidogenic enzymes such as CYP11A1, CYP17A1, HSD3B and StAR, but in contrast to the control group, this group maintained a slightly low expression level. Inspiringly, at 3 weeks after grafting, the iLCs group strongly expressed these steroidogenic biomarkers, and in particular, the expression levels of HSD3B and StAR were very close to those of the controls, while the model and HFFs groups demonstrated nearly negative expression of these biomarkers. Frustratingly, expression levels of these biomarkers in the iLCs group decreased significantly at 5 weeks after transplantation, and in particular, the expression levels of CYP11A1, CYP17A1 and StAR were lower. As steroidogenic enzymes are key for testosterone synthesis,^38^, ^39^, ^40^ the down‐regulated expression of these biomarkers may be a direct factor leading to the decline of testosterone levels over 3 weeks after grafting. Regarding the above phenomenon, the new niche for the implanted iLCs may play a vital role**.** 41, 42 In the early stage of transplantation, the cells may need to adapt to the new environment, and therefore, they maintain a slightly low activity. The activity of these implanted iLCs began to increase gradually approximately one week later. However, as time progresses, some adverse factors in vivo may accelerate cell ageing and functional decline such as oxidative stress, immune responses and so on. In addition, loss in the steroidogenic function over time may be related to two important factors: loss in cell viability due to poor circulation within the Matrigel and the absence of the normal testicular niche cells such as Sertoli cells or myoid cells. In particular, the latter factor may play a key role. Therefore, in the future, there is a need to maintain an appropriate niche for the high activity of implanted iLCs.

Next, to compare the characteristics of post‐transplanted iLCs with pre‐transplanted iLCs, we isolated iLCs by Corning cell recovery solution from the implanted Matrigel at 6 weeks after grafting. The isolated cells were cultured for 3 days and showed abnormal cell size and shape compared with the pre‐transplanted iLCs, which indicated that these isolated iLCs may be in an ageing state. Ageing cells will undergo decreased function as well as morphological changes.^43^, ^44^, ^45^ Subsequently, we observed that the iLCs isolated from the implanted Matrigel still weakly secreted testosterone, but adding hCG failed to significantly promote the secretion of testosterone compared with the pre‐transplanted iLCs. In addition, expression levels of key steroidogenic enzymes such as CYP11A1 and CYP17A1 significantly decreased compared with pre‐transplanted iLCs. The data indicate that with prolonged cell transplantation, the function of implanted cells gradually declines, and cell ageing may be another major reason for this effect. Therefore, in the future, it is worth studying how to delay the ageing of transplanted cells in vivo.

In conclusion, to our knowledge, we are the first to successfully reprogram human fibroblasts into testosterone‐producing Leydig‐like cells expressing Leydig cell lineage‐specific markers by this new strategy, which was very encouraging. Furthermore, when the purified iLCs were transplanted into the fibrous capsule of castrated rats, the implanted iLCs were capable of surviving and partially restoring serum testosterone levels, and they could weakly mimic the role of adult Leydig cells in the hypothalamic‐pituitary‐gonadal axis for a short period. Therefore, this study may be valuable for male hypogonadism in the future.

## CONFLICT OF INTERESTS

The authors declare no conflict of interest.

## AUTHOR CONTRIBUTIONS

HH and JS conceived and designed this study; HH, LZ, JZ and YPH performed the research; HH and LZ analysed the data; XYX contributed materials; HH and JS wrote the paper. All authors read and approved the content.

## Data Availability

The data in the current study are available from the corresponding authors on reasonable request.
