# Supplementary figures and images for: Leydig‐like cells derived from reprogrammed human foreskin fibroblasts by CRISPR/dCas9 increase the level of serum testosterone in castrated male rats
Source: J Cell Mol Med. 2020 Mar 11;24(7):3971–81. doi: 10.1111/jcmm.15018 (PMC7171312; doi:10.1111/jcmm.15018)

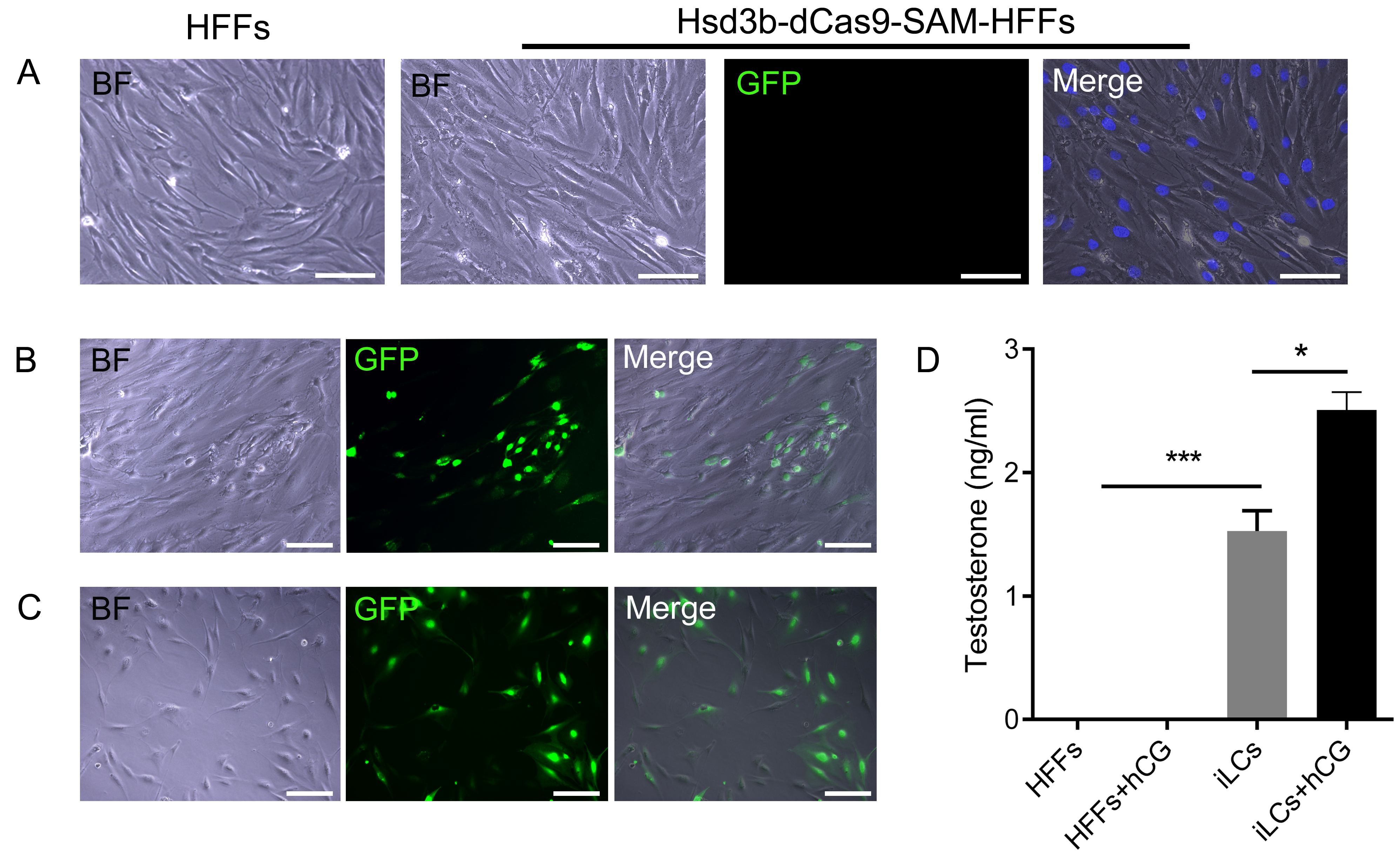

Supplement: Supplementary file 1 [file JCMM-24-3971-s001.tif]
