# Supplementary material for: Leydig‐like cells derived from reprogrammed human foreskin fibroblasts by CRISPR/dCas9 increase the level of serum testosterone in castrated male rats
Source: J Cell Mol Med. 2020 Mar 11;24(7):3971–81. doi: 10.1111/jcmm.15018 (PMC7171312; doi:10.1111/jcmm.15018)
Supplement: Supplementary file 2 [file JCMM-24-3971-s002.docx]

**Table S1. Three sgRNA sequences used for each target gene promoter.**

| **Target promoter** | **Forward primer** | **Reverse primer** |
| --- | --- | --- |
| ***Nr5a1*** | CACCGTAGCCATTCACAAGGAGAG | AAACCTCTCCTTGTGAATGGCTAC |
|  | CACCGGGACCCCACAGAGGCGGGT | AAACACCCGCCTCTGTGGGGTCCC |
|  | CACCGGCCCGCTGTGGGAGCCCAG | AAACCTGGGCTCCCACAGCGGGCC |
| ***Gata4*** | CACCGAGGTCACCTTCTTCTCTAC | AAACGTAGAGAAGAAGGTGACCTC |
|  | CACCGCGCAGCGGCGGGCGAGGGGC | AAACGCCCCTCGCCCGCCGCTGCGC |
|  | CACCGACAGGAAGGGGGGGCGGGGA | AAACTCCCCGCCCCCCCTTCCTGTC |
| ***Dmrt1*** | CACCGCACGGCTCTGGTCTTGACCT | AAACAGGTCAAGACCAGAGCCGTGC |
|  | CACCGTGCACCGTGCTTTGGCAACC | AAACGGTTGCCAAAGCACGGTGCAC |
|  | CACCGAGAGCGGCCTGGGAATCGCG | AAACCGCGATTCCCAGGCCGCTCTC |
